# Supplementary material for: Phonon-assisted up-conversion photoluminescence of quantum dots
Source: Nat Commun. 2021 Jul 13;12:4283. doi: 10.1038/s41467-021-24560-4 (PMC8277828; doi:10.1038/s41467-021-24560-4)
Supplement: Supplementary file 1 — Supplementary Information [file 41467_2021_24560_MOESM1_ESM.pdf]

Supplementary Information for

Phonon-assisted up-conversion photoluminescence of quantum dots

Zikang Ye[†], Xing Lin[†], Na Wang, Jianhai Zhou, Meiyi Zhu, Haiyan Qin[*] and Xiaogang Peng[*]

* Corresponding to: hattieqin@zju.edu.cn and xpeng@zju.edu.cn

† These authors contributed equally to this work.

Supplementary Methods:

Chemiscals. Myristic acid (98%), stearic acid (>90%), lauric acid (99.5%), cadmium oxide (CdO, 99.998%), selenium power (Se, 200 mesh, 99.999%), 1-octadecene (ODE, 90%), oleic acid (99% or 90%), sulfur powder (S, 99.98%), oleylamine (>98%) and polymethyl methacrylate (PMMA, >98.0%) were purchased from either Aldrich or Alfa-Aesar. Tributylphosphine (TBP) was purchased from Acros. Cyclopentane (>98.0%) were purchased from TCI. Toluene, methanol, chloroform, acetone, and acetonitrile were obtained from Sinopharm Reagents. All chemicals were used directly without any further purification unless otherwise stated.

Synthesis of CdSe core QDs. CdSe core QDs with the first absorption peak at 570 nm were synthesized according to the published procedures[1]. A brief description is as follows. Se-suspension was prepared by dispersing Se powder (0.15 mmol) in ODE (3 mL) by sonication for 5 min. In a typical synthesis, CdO (0.2 mmol) and myristic acid (0.45 mmol) were loaded into a 25 mL three-neck flask with 4 mL of ODE. After being stirred and argon-bubbled for 10 min, the mixture was heated to 290 °C to obtain a colourless solution. After the mixture was cooled to 250 °C, 1 mL of Se-suspension was injected quickly into the flask, which further brought down the temperature to 220 °C. The reaction temperature was kept at 240 °C for further growth. After growth for 5 min, another Se-suspension prepared by dispersing Se powder (0.3 mmol) in 2 mL ODE and 1 mL oleic acid was loaded into a syringe and dropwise-added into the reaction flask at 0.015 mL/min. Shaking of the suspension in the syringe was needed every 5 minutes to keep its uniformity. After one dose of Se-suspension was added, the reaction solution was allowed to react for 5 min. This

reaction cycle, that is, addition of one dose of Se-suspension and reacting for 5 min, was continued until the CdSe QDs with targeted size were obtained.

Purification of CdSe core QDs. For purification of CdSe core QDs, a mixed solution of acetone, chloroform, and methanol (volume ratio 1:1:1) was prepared as the precipitation solution. The crude reaction solution (1−1.5 mL) and the precipitation solution (2 mL) were loaded into a 4 mL vial in succession. After being heated to ∼50 °C, the vial was immediately centrifuged at 1700 g for ∼20 s. The supernatant was removed quickly. The nanocrystal precipitate was then dissolved in ∼0.5 mL of toluene, into which 2 mL of precipitation solution was added again for another cycle of purification. This purification procedure was repeated for another two times.

Epitaxial growth of CdS to synthesize CdSe/CdS core/shell QDs. S-ODE solution was prepared by dissolving sulfur powder (1 mmol) in ODE (10 mL) by sonication. In a typical synthesis, CdO (0.5 mmol) and myristic acid (1.1 mmol) (or 1.15 mmol of lauric acid) were loaded into a 25 mL three-neck flask with 3.5 mL of ODE. After being stirred and argon-bubbled for 10 min, the mixture was heated to 290 °C to become a colourless solution. The mixture was then cooled below 150 °C. Purified CdSe core QDs dissolved in ODE were injected into the reaction solution. The new mixture was heated to 250 °C under argon flow. One dose of S-ODE was loaded into a syringe and dropwise added into the reaction flask at 0.015 mL/min. After one dose of S-ODE was added, the reaction solution was allowed to react for 2 min. One dose of oleic acid (0.2 mmol) was added into the flask at 0.2 mL/min, followed by 2 min stirring. For the second cycle, the reaction durations were both 5 min after additions of S-ODE and oleic acid. This reaction cycle, addition of one dose of S-ODE and reacting for 5 min followed by addition of one dose of oleic acid and reacting for

another 5 min, was repeated until the targeted CdSe/CdS core/shell QDs were obtained. For the synthesis of high-quality CdSe/CdS core/shell QDs with shell thickness being 7 to 8 monolayers (photoluminescence peak position being 630 nm), the ratio of the total amount of oleic acid to myristic acid (or lauric acid) was between 3:1 and 4:1.

Ligand exchange. Mixture of 1 mL ODE and 2 mL oleylamine in a three-neck flask was heated to 200 °C under argon flow. 0.1 mL of TBP was injected into this mixture. Purified core/shell QDs coated with carboxylate ligands were dissolved in 0.3 mL of ODE and injected into the flask. The reaction mixture was maintained at 200°C for 5−10 min to complete the ligand exchange.

Determination of photoluminescence quantum yields (PLQY) of QDs. The absolute photoluminescence quantum yield of the quantum dots excited at 450 nm was exclusively determined using an integral sphere system (Supplementary Fig. 5a). Both the absorption and emission were measured according to the irradiance of the light exiting form the integral sphere. The integral sphere system was calibrated with a standard light source. The accuracy and reproducibility of the system are confirmed by measuring the quantum yields of organic dyes with known quantum yields (Rhodamine-6G and Nile Red) with the methods described in the corresponding literatures[2,3].

According to the definition of photoluminescence quantum yield:

$$N_{\mathrm{PL}} = N_{\mathrm{abs}} \times \phi_{\mathrm{PL}} \quad \text{(Eq. S1)}$$

, where N_{PL}, N_{abs}, ϕ_{PL} are photon number of photoluminescence, number of excitation light absorbed by the sample and photoluminescence quantum yield of the sample.

$$N_{\mathrm{PL}} = (N_0 - N_{\mathrm{t}}) \times \phi_{\mathrm{PL}} \quad \text{(Eq. S2)}$$

N_0 denotes total photon number of excitation light irradiating on the sample and N_{t} denotes photon number of residual excitation light. Considering the detection efficiency of photoluminescence and residual excitation light to be $\eta_{\mathrm{PL,det}}$ and $\eta_{\mathrm{t,det}}$, one gets:

$$\frac{N_{\mathrm{PL,det}}}{\eta_{\mathrm{PL,det}}} = \left(N_0 - \frac{N_{\mathrm{t,det}}}{\eta_{\mathrm{t,det}}}\right) \times \phi_{\mathrm{PL}} \quad \text{(Eq. S3)}$$

, where $N_{\mathrm{PL,det}}$ and $N_{\mathrm{t,det}}$ are the photon number of detected photoluminescence and detected residual excitation light. Since the system had been calibrated with a standard light source, the detection efficiencies over the whole spectrum are calibrated to be identical.

$$\eta_{\mathrm{PL,det}} = \eta_{\mathrm{t,det}} \equiv \eta_{\mathrm{det}} \quad \text{(Eq. S4)}$$

Therefore, Eq. S3 can be simplified as:

$$N_{\mathrm{PL,det}} = -\phi_{\mathrm{PL}} \times N_{\mathrm{t,det}} \times +N_0 \times \eta_{\mathrm{det}} \times \phi_{\mathrm{PL}} \quad \text{(Eq. S5)}$$

For quantum dots with different concentration and the same volume, taking $N_{\mathrm{t,det}}$ and $N_{\mathrm{PL,det}}$ as x and y axes, the quantum yield (ϕ_{PL}) can be determined from the absolute slope based on a linear fitting.

During measurement, an 1 mW light-emitting diode (LED) with 450 or 532 nm peak wavelength and 20 nm band-width was used as the excitation light source. The excitation light was directed to excite the sample. 200 μL of sample solutions with different

concentrations along with a blank solvent were successively filled into glass tubes with 5 mm diameter and 40 mm length which were then placed inside an Ocean Optics FOIS-1 integrating sphere. An Ocean Optics QE65000 spectrometer was used to record the photon counts of the photoluminescence and the residual excitation light. The system was calibrated with an Ocean Optics HL-3-CAL standard light source. A schematic diagram of the setup was shown in Supplementary Fig. 5a.

The photoluminescence quantum yield of Nile Red and Rhodamine 6G were measured to confirm the accuracy of the system with the methods reported in literatures. Nile Red-acetonitrile solutions with concentration ranging from 0.75 to 15 μmol/L and Rhodamine 6G-ethanol solutions with concentration ranging from 10 to 200 μmol/L were prepared. The solutions along with blank solvents were degassed with Argon before measurements. The Nile Red solutions were excited with the 532 nm LED and the Rhodamine 6G solutions were excited with the 450 nm LED. All measurements were carried out at room temperature and ambient atmosphere. The reported photoluminescence quantum yields of Rhodamine-6G and Nile Red are 94%[3] and 78%[2]. The average value of photoluminescence quantum yields of Rhodamine-6G and Nile Red over three parallel tests were 0.92 and 0.79 respectively (Supplementary Fig. 6). The standard derivations were both 0.01. The relatively lower value of photoluminescence quantum yield of Rhodamine-6G can be attributed to sample degeneration. The experimental conditions including solvent, temperature and concentration were exactly the same as those reported in the literatures.

For the measurement of absolute photoluminescence quantum yields of quantum dots excited at 450 and 532 nm, quantum dot solutions with different concentrations were prepared by dissolving the quantum dots into toluene. The results were shown in

Supplementary Fig. 7.

The UCPL quantum yields of the quantum dots excited with 638 nm was determined with a relative method (Supplementary Fig 5b), in which absorbance was measured by a power meter and photoluminescence was measured by a spectrometer. By irradiating the sample with 638 and 450 nm laser respectively, we can determine the ratio of the quantum yield excited at 638 nm to the quantum yield excited at 450 nm. Thus, the UCPL quantum yield excited at 638 nm was determined by taking the quantum yield excited at 450 nm as reference. The accuracy of this method was confirmed by measuring both the absolute and relative quantum yields of the sample excited at 532 nm. The results are 0.97 and 0.98 (Supplementary Fig. 7 & 8) respectively. The reason why we use a relative method to determine the UCPL quantum yield is that the absorbance of the sample excited at sub-bandgap wavelength is so low that the residual excitation light is much stronger than the photoluminescence. Furthermore, in terms of spectrum recording, sub-bandgap excitation and emission overlap with each other heavily. These facts make it difficult to accurately determine the absolute UCPL quantum yield of the sample using an integrating sphere system. In the relative method, collecting emission signal perpendicular to the excitation light minimizes the interference of the residual excitation light.

During experiments, three samples of QD solution were filled into 1-cm optical path quartz cuvettes and irradiated by one of the 450, 532, 638 nm narrowband lasers respectively (Supplementary Fig 5b). The absorbance values of the samples were kept similar to exclude the influence of inner-filter and re-absorption by adjusting the concentrations of the solutions (the quantum yield remains identical among a large range of concentration, see Supplementary Fig. 7). The concentration of QD solution excited at 638 nm was the highest

due to small absorption cross-section at the excitation wavelength. The excitation lights from different lasers were shaped to keep identical spatial profile using an optical fibre and an iris. The excitation lights irradiate the samples on the edge of the cuvettes to minimize the influence of re-absorption. The photoluminescence was collected perpendicular to the excitation light beam at the side of the cuvette through an optical fibre and recorded with an Ocean optics QE65000 spectrometer. The refractive indexes of the most concentrated and most dilute samples were 1.4860 and 1.4932 respectively measured by an Abbe refractometer at room temperature, which are very close to each other and match well with the refractive index of the quartz cuvette (1.4584). The reflectivity on the interface of cuvette and all solutions are smaller than 0.1%, which can be ignored. The relative PLQY excited at a wavelength of λ other than 450 nm was calculated as

$$\phi_{\mathrm{PL}}(\lambda) = \phi_{\mathrm{PL}}(450\ \mathrm{nm}) \times \frac{n^2(\lambda)}{n^2(450)} \times \frac{N_{\mathrm{PL}}(\lambda)}{N_{\mathrm{PL}}(450\ \mathrm{nm})} \times \frac{N_{\mathrm{abs}}(450\ \mathrm{nm})}{N_{\mathrm{abs}}(\lambda)} \qquad \text{(Eq. S6)}$$

, where $N_{\mathrm{PL}}(\lambda)$ and $N_{\mathrm{abs}}(\lambda)$ are the integral photon counts of photoluminescence excited at the wavelength of λ and the integral photon counts of the excitation light absorbed at the wavelength of λ respectively. $n(\lambda)$ is the refractive index at wavelength of λ. $N_{\mathrm{PL}}(\lambda)$ was recorded by a calibrated Ocean Optics QE65000 spectrometer. $N_{\mathrm{abs}}(\lambda)$ was calculated from the power differences measured by a Thorlabs S121C power meter placing in front of and behind the sample. The power meter was placed at the path of laser and 0.2 m away from the cuvette when measuring the laser power propagating through the sample to avoid the interference from the emission light. The extinction of excitation light by cuvette and solvent were deducted. The photoluminescence spectra normalized by the photon counts

of the absorbed excitation light $N_{\mathrm{abs}}(\lambda)$ was shown in Supplementary Fig. 8. The schematic diagram of the setup was shown in Supplementary Fig. 5b.

Temperature calibration and determination methods in optical cooling experiments. In order to measure the temperature changes with high sensitivity, a novel temperature determination method based on the special geometry structure of the 'QD-thermometer' was developed. According to the principle of thermal expansion of liquids, the volume change of the QD solution during laser irradiation can be used to determine the overall temperature change of the solution. Since the inner diameter of the capillary tube is very small (only 0.16 mm), faint temperature change can drive the liquid-air interface to move a relatively long distance in the narrow capillary. The temperature resolution of this method can be as high as 20 mK. The movement of the liquid-air interface position was recorded as a sequence of images taken by digital camera with high spatial resolution (Supplementary Fig. 22).

The temperature calibration was done by measuring the liquid level at different temperature with a water bath (Supplementary Fig. 19). A marker with sharp edges was attached on the capillary tube, working as a length basis. The length ratio of the liquid column (L) and the marker (L_{m}) would not be affected by neither the distance between tube and camera nor their relative orientation. Therefore, $\frac{L}{L_{\mathrm{m}}}$ was utilized as a reliable indicator for temperature calibration and determination. During temperature calibration, the bulb reservoir partially filled with QD solution or solvent was immersed into a water bath with designated temperatures. The temperature was detected by a calibrated LINI-T UT323 thermometer

with its thermocouple immersed in the water bath and close to the bulb reservoir. We use a camera to take photos of the capillary tube. $\frac{L}{L_{\mathrm{m}}}$ at different temperatures were acquired from the photos via image processing. The calibration results for QD solution and control specimen are shown in Supplementary Fig. 20.

During optical cooling experiments, the temporal evolution of $\frac{L}{L_{\mathrm{m}}}$ was recorded with a Canon 5D IV digital camera with a CMOS chip containing 6720×4480 pixels. The camera was firmly fix on the optical table. Photographs were taken every 5 to 10 s. The time sequence of temperature change of QD sample and control specimen under laser irradiation at 671 nm are shown in Fig 4c. The temperature changes at other wavelengths are shown in Supplementary Fig. 21. Three parallel experiments were carried out for both QD sample and control specimen (Supplementary Fig. 23) to verify the repeatability and to give the error bars marked in Fig. 4b. The values of both positive and negative error bars are the sums of standard deviations of the temperature changes of the QD sample and the control specimen at each excitation condition.

Modelling of the optical cooling/heating process

(a) Liquid expansion

The cubic expansion coefficient (α) is defined as:

$$\alpha = \frac{1}{V}\left(\frac{\partial V}{\partial T}\right)_P \qquad \text{(Eq. S7)}$$

, where V is the volume of the liquid sample, T is temperature and P is pressure. When pressure is a constant ($dP \equiv 0$),

$$\alpha = \frac{1}{V}\frac{dV}{dT} \qquad \text{(Eq. S8)}$$

The volume change of the liquid (ΔV) can be expressed as:

$$\Delta V = V_T - V_0 = [\exp(\alpha \Delta T) - 1]V_0 \qquad \text{(Eq. S9)}$$

, where V_T and V_0 are the final and initial volumes. Based on the geometry of the solution container, ΔV can be expressed as the product of the sectional area of capillary tube (A_c) and the change of the liquid level (ΔL):

$$\Delta V = A_\mathrm{c}\Delta L = A_\mathrm{c}L_\mathrm{m}\Delta\left(\frac{L}{L_\mathrm{m}}\right) \qquad \text{(Eq. S10)}$$

, where L is the length of the liquid column and L_m is the length of the marker (Supplementary Fig. 19a). According to Eq. S9 and Eq. S10,

$$\Delta\left(\frac{L}{L_\mathrm{m}}\right) = [\exp(\alpha \Delta T) - 1]\frac{V_0}{A_\mathrm{c}L_\mathrm{m}} \qquad \text{(Eq. S11)}$$

When $\alpha \Delta T \ll 1$,

$$\Delta\left(\frac{L}{L_\mathrm{m}}\right) \approx \frac{V_0 \alpha}{A_\mathrm{c}L_\mathrm{m}}\Delta T \qquad \text{(Eq. S12)}$$

The cubic expansion coefficient of the QD solution approximately equals to the cubic expansion coefficient of toluene, which is 1.05×10^{-3} K^{-1} at 25 ℃ [4]. The geometry parameters of the solution container are: $L_\mathrm{m} = 6$ mm; $R_\mathrm{r} = 1$ mm (radius of the bulb

reservoir); $R_{\mathrm{c}} = 0.08$ mm (radius of the capillary); $L_{\mathrm{r}} = 10$ mm (length of the bulb reservoir); $L_{\mathrm{c}} = 30$ mm (length of the liquid column); $V_0 = \pi R_{\mathrm{r}}^2 L_{\mathrm{r}} + \pi R_{\mathrm{c}}^2 L_{\mathrm{c}} = 32$ mm^3; $A_{\mathrm{c}} = \pi R_{\mathrm{c}}^2 = 0.02$ mm^2.

According to the above parameters, Eq. S12 can be simplified as:

$$\Delta\left(\frac{L}{L_{\mathrm{m}}}\right) \approx 0.279\Delta T \qquad \text{(Eq. S13)}$$

, which matches the experimental results (Supplementary Fig. 20) well.

(b) Heat capacity

The heat capacity of the sample (C_p) was the sum of the heat capacity of solvent ($C_{p,\mathrm{tol}}$), quartz tube ($C_{p,\mathrm{q}}$), quantum dots ($C_{p,\mathrm{QD}}$) and additional ligands ($C_{p,\mathrm{ligand}}$). The first two contribute most of the heat capacity of the sample:

$$C_p = C_{p,\mathrm{tol}} + C_{p,\mathrm{q}} + C_{p,\mathrm{QD}} + C_{p,\mathrm{ligand}} \approx C_{p,\mathrm{tol}} + C_{p,\mathrm{q}} \qquad \text{(Eq. S14)}$$

The heat capacity of the toluene was calculated to be 0.0473 J/K according to the following equation:

$$C_{p,\mathrm{tol}} = C_{p,\mathrm{tol,m}} n_{\mathrm{tol}} = C_{p,\mathrm{tol,m}} \frac{\rho_{\mathrm{tol}} V_{\mathrm{tol}}}{M_{\mathrm{tol}}} \qquad \text{(Eq. S15)}$$

, where $C_{p,\mathrm{tol,m}}$ is the molar heat capacity of toluene[4], which is 157.3 J mol^{-1} K^{-1}, $\rho_{\mathrm{tol}} =$ 0.866 gcm^{-3} is the density of toluene and $M_{\mathrm{tol}} = 92.1$ gmol^{-1} is the molar mass of toluene. The volume of toluene (V_{tol}) is close to the volume of the solution (V_0), which is 32 mm^3.

The heat capacity of the quartz tube was calculated to be 0.121 J/K according to the

following equation:

$$C_{p,\mathrm{q}} = C_{p,\mathrm{q,m}} n_{\mathrm{tol}} = C_{p,\mathrm{q,m}} \frac{m_{\mathrm{q}}}{M_{\mathrm{q}}} \qquad \text{(Eq. S16)}$$

, where $C_{p,\mathrm{q,m}}$ is the molar heat capacity of fused quartz[4], which is 44.77 J mol^{-1} K^{-1}. $m_{\mathrm{q}} =$ 0.16 g is the mass of the quartz tube. $M_{\mathrm{q}} = 60.08$ gmol^{-1} is the molar mass of quartz (SiO_2). Thus, the heat capacity of sample and solvent were calculated to be 0.168 J/K.

(c) Thermal load

The thermal load or heat transfer power (P_{load}) is another key parameter to model the cooling/heating process. The heat transfer occurs in three ways: conduction, convection and thermal radiation. The total thermal load can be expressed as the sum of three thermal loads terms corresponding to these three processes:

$$P_{\mathrm{load}} = P_{\mathrm{cond}} + P_{\mathrm{conv}} + P_{\mathrm{rad}} \qquad \text{(Eq. S17)}$$

When the temperature change is small, the thermal load scale linearly with temperature difference between sample and surrounding heat bath (ΔT):

$$P_{\mathrm{load}} \approx k_{\mathrm{cond}}\Delta T + k_{\mathrm{conv}}\Delta T + k_{\mathrm{rad}}\Delta T = (k_{\mathrm{cond}} + k_{\mathrm{conv}} + k_{\mathrm{rad}})\Delta T \qquad \text{(Eq. S18)}$$

The ratio of P_{load} and ΔT is defined as k_{load}, which consist of contribution from conduction (k_{cond}), convection (k_{conv}) and thermal radiation (k_{rad}):

$$k_{\mathrm{load}} \equiv k_{\mathrm{cond}} + k_{\mathrm{conv}} + k_{\mathrm{rad}} \qquad \text{(Eq. S19)}$$

Specially, thermal load delivered by a net absorption of thermal radiation is:

$$P_{\mathrm{rad}} = \sigma\varepsilon A(T_{\mathrm{S}}^4 - T_{\mathrm{R}}^4) \approx 4\varepsilon\sigma A T_{\mathrm{R}}^3 \Delta T \qquad \text{(Eq. S20)}$$

, where σ is Stefan-Boltzmann constant, ε is the emissivity of the sample surface, A is sample's surface area, T_{S} and T_{R} are temperature of sample and surrounding thermal bath. $\Delta T \equiv T_{\mathrm{S}} - T_{\mathrm{R}}$. Using the parameters: $\sigma = 5.67\times10^{-8}$ $\mathrm{Wm^{-2}K^{-4}}$, $\varepsilon = 0.93$ at room temperature, $A = \pi R_{\mathrm{r}}^2 + 2\pi R_{\mathrm{r}} L_{\mathrm{r}} + 2\pi R_{\mathrm{c}} L_{\mathrm{c}}$ =81 $\mathrm{mm^2}$ and T_{R} =295.5 K, the thermal load delivered by thermal radiation for unit temperature difference (k_{rad}) is estimated as 0.44 mW/K. This is the lowest limit of k_{load}.

(d) Cooling and heating kinetics:

The temperature kinetics is governed by the equation:

$$\frac{dT}{dt} = -\frac{1}{C_p} k_{\mathrm{load}}(T - T_{\mathrm{R}}) + \frac{1}{C_p} P_{\mathrm{in}} \qquad \text{(Eq. S21)}$$

, where P_{in} is the heat generation power induced by laser irradiation. With the initial condition $T(t=0) = T_{\mathrm{R}}$, we can solve the Eq S21 and get:

$$T(t) = T_{\mathrm{R}} + \frac{P_{\mathrm{in}}}{k_{\mathrm{load}}}\left(1 - e^{-\frac{k_{\mathrm{load}}}{C_p}t}\right) \qquad \text{(Eq. S22)}$$

, which describes the temperature increasing process when the laser is turned on. Maximum temperature change at steady state is given by $\Delta T_{\mathrm{max}} = \frac{P_{\mathrm{in}}}{k_{\mathrm{load}}}$.

With the initial condition $T(t=0) = T_{\mathrm{R}} + \Delta T_{\mathrm{max}}$ and set $P_{\mathrm{in}} = 0$, we can solve the Eq. S21 and get:

$$T(t) = T_{\mathrm{R}} + \Delta T_{\mathrm{max}} e^{-\frac{k_{\mathrm{load}}}{C_p}t} \qquad \text{(Eq. S23)}$$

, which decries the temperature decreasing process when laser is turned off. Both the temperature increasing and decreasing process is governed by an exponential function with time constant of

$$\tau = \frac{C_p}{k_{\text{load}}} \quad \text{(Eq. S24)}$$

Based on calculations in section (b) and (c), considering thermal load only delivered by thermal radiation, the time constant τ is estimated as 0.168/0.00044≈381s while the experimental values are around 110s (Fig 4c). The discrepancy may come from two points:

1, The temperature may not be uniform over the whole sample, thus the effective heat capacity is smaller than estimation;

2, We underestimate the thermal load due to the non-negligible contact of sample with the holder and the residual air in the chamber.

(e) Excitation wavelength-dependence

For the control specimen under laser irradiation, the power gain ($P_{\text{in,b}}$) is the product of the incident laser power (P_0) and the absorption ratio of the incident laser ($\eta_{\text{abs,b}}$).

$$P_{\text{in,b}} = P_0 \eta_{\text{abs,b}} \quad \text{(Eq. S25)}$$

Inserting Eq. S25 into Eq. S22, the temperature change of the control specimen (ΔT_{b}) can be expressed as:

$$\Delta T_{\text{b}}(t) = \frac{P_0 \eta_{\text{abs,b}}}{k_{\text{load}}} \left(1 - e^{-\frac{k_{\text{load}}}{C_p} t}\right) \quad \text{(Eq. S26)}$$

The maximum temperature change $\Delta T_{\mathrm{b,max}}$ was acquired when the system was in thermal equilibrium.

$$\Delta T_{\mathrm{b,max}} = \frac{P_0 \eta_{\mathrm{abs,b}}}{k_{\mathrm{load}}} \qquad \text{(Eq. S27)}$$

For QD solution under laser irradiation, the temperature change rate $P_{\mathrm{in,s}}$ can be expressed as:

$$P_{\mathrm{in,s}} = P_0[\eta_{\mathrm{abs,s}}(1 - \eta_{\mathrm{p}}\eta_{\mathrm{qy}}) + \eta_{\mathrm{abs,b}}] \qquad \text{(Eq. S28)}$$

, where $\eta_{abs,s}$ is the absorption ratio of the quantum dots, acquired by absorption spectrum (the band edge absorptions are acquired by an exponential fitting to the absorption spectrum band-tail). η_{qy} is the quantum yield of QD. $\eta_{\mathrm{p}} = \frac{\lambda_{\mathrm{ex}}}{\overline{\lambda_{\mathrm{PL}}}}$ is the photon energy conversion efficiency, where λ_{ex} is the wavelength of the excitation photons and $\overline{\lambda_{\mathrm{PL}}}$ is the average wavelength of photoluminescence. By inserting Eq. S28 into Eq. S22, the temperature change of QD sample (ΔT_{s}) can be expressed as:

$$\Delta T_{\mathrm{s}}(t) = \frac{P_0[\eta_{\mathrm{abs,s}}(1-\eta_{\mathrm{P}}\eta_{\mathrm{qy}})+\eta_{\mathrm{abs,b}}]}{k_{\mathrm{load}}}\left(1 - e^{-\frac{k_{\mathrm{load}}}{C_p}t}\right) \qquad \text{(Eq. S29)}$$

The maximum temperature change $\Delta T_{\mathrm{s,max}}$ was acquired when the system was in thermal equilibrium.

$$\Delta T_{\mathrm{s,max}} = \frac{P_0[\eta_{\mathrm{abs,s}}(1-\eta_{\mathrm{P}}\eta_{\mathrm{qy}})+\eta_{\mathrm{abs,b}}]}{k_{\mathrm{load}}} \qquad \text{(Eq. S30)}$$

The temperature difference between QD solution and control specimen per unit excitation power at different excitation wavelength is:

$$\frac{\Delta T_{\mathrm{s,max}} - \Delta T_{\mathrm{b,max}}}{P_0} = \frac{[\eta_{\mathrm{abs,s}}(1-\eta_{\mathrm{P}}\eta_{\mathrm{qy}})]}{k_{\mathrm{load}}} \quad \text{(Eq. S31)}$$

The temperature difference between QD solution and control specimen scales linearly with the excitation power, which is consistent with the power dependent results in Fig. 4d. Wavelength-dependent results in Fig. 4b are fitted with Eq. S31 with η_{qy} and $\eta_{\mathrm{abs,s}}/k_{\mathrm{load}}$ as free parameters. η_{qy} was fitted as 95.7 %, which is lower than the quantum yield of dilute solution. It can be attributed to re-absorption, energy transfer and adsorption of QDs on the inner wall of the capillary tube.

Calculation of phonon-assisted absorption probability

The phonon population (N_{p}) of a specific mode obeys Bose-Einstein statistics, as

$$N_{\mathrm{p}} = \frac{1}{e^{E_{\mathrm{p}}/k_{\mathrm{B}}T} - 1} \quad \text{(Eq. S32)}$$

, where E_{p} is the phonon energy (25meV for the LO phonon energy of CdSe), k_{B} is the Boltzmann constant and T is temperature. The probability of the electronic transition coupling simultaneously with n phonons should be in proportion to the nth power of the phonon population:

$$P \propto (N_{\mathrm{p}})^n = \left(\frac{1}{e^{E_{\mathrm{p}}/k_{\mathrm{B}}T} - 1}\right)^n \quad \text{(Eq. S33)}$$

The calculated absorption spectrum in Fig. 5b comes from Eq. S33. The coupled phonon number n is calculated as $\frac{E_{1\mathrm{S}} - E_{\mathrm{ex}}}{E_{\mathrm{LO}}}$, where $E_{1\mathrm{S}}$, E_{ex} and E_{LO} are the energies of the 1st

excitonic absorption peak, the excitation photon and the longitudinal optical phonon of CdSe.

Based on Eq. S33, the excitation transition probability at 660 nm with 5 phonons involved is 6.8% of that at 620 nm. The experimental result is 0.9%. The discrepancy should be attributed to the Franck-Condon factor (the overlap integral of the vibrational wave functions of the initial and final states) which decreases with increasing of the coupled phonons for materials with small Huang-Rhys factors, such as quantum dots.

Supplementary Discussion:

Size inhomogeneity induced spectral difference between UCPL and DCPL in ensemble level.

We attribute the slight red-shifting and narrowing of the UCPL spectrum compared with the corresponding DCPL spectrum in Fig. 1a to the minor degree of size inhomogeneity of QDs. QDs with slightly larger sizes among the ensemble have relatively red-shifted absorption and photoluminescence spectra due to their weaker quantum confinement. Therefore, excitation at the falling edge ($dA/d\lambda < 0$, where A is the absorbance and λ is the absorption wavelength) of the absorption spectrum of an ensemble QD sample will selectively excite the larger particles in it, resulting in red-shifted photoluminescence. Similarly, excitation at the rising edge ($dA/d\lambda > 0$)) of the absorption spectrum shall result in slight blue-shifted photoluminescence.

Supplementary Fig. 3 presents a correlation between the photoluminescence peak position shift and the gradient of absorption spectrum ($dA/d\lambda$) at some specific excitation wavelengths (λ) of ensemble CdSe QDs with relatively poor dispersity. It shows that these two variables not only have the opposite signs but also have a good negative correlation.

Although the UCPL spectrum reported in Fig. 1a doesn't perfectly overlap the DCPL spectrum in the ensemble level, the difference is much smaller than those reported in the previous literatures[5-7] due to much better size homogeneity of the sample. Moreover, the UCPL and DCPL spectra can be well-overlapped in single-dot level as shown in Fig. 3b, which further confirmed that the difference observed in the ensemble level is caused by the slightly imperfect size distribution of the QDs.

Dependence of core size and shell thickness on the up-conversion capability of quantum dots

The optical up-conversion capability of QDs with unity quantum yield depends on the absorption ability of low-energy photons and the energy discrepancy between emitted and absorbed photons. It's obvious that QDs with large intrinsic photoluminescence band-width and small Stokes-shift are favored for optical up-conversion. However, these two factors follow the same trend with core size and shell thickness for CdSe/CdS core/shell QDs[1], requiring a trade-off for selecting the ideal sample with optimized core size and shell thickness. In order to quantitatively evaluate the up-conversion capability of QDs, we define an up-conversion capability index η as the ratio of the absorbance at energy of 50 meV (the energy of twice the longitudinal optical phonons of CdSe) lower than the average photoluminescence energy ($A_{\langle PL\rangle-2\mathrm{LO}}$, Supplementary Fig. 26a, orange line) and the absorbance at the first excitonic absorption peak ($A_{\mathrm{abs\ peak}}$, Supplementary Fig. 26a, blue line), that is

$$\eta = A_{\langle PL\rangle-2\mathrm{LO}}/A_{\mathrm{abs\ peak}} \qquad \text{(Eq. S34)}$$

Supplementary Fig. 26c presents the core size and shell thickness dependence of η calculated based on the experimental photoluminescence and absorption spectra of a series of monodisperse CdSe/CdS core/shell QDs[1]. Maximum value of η is found in QDs with small cores and thin shells, indicating that the influence of their large photoluminescence band-widths overcome the relatively large Stokes shifts. However, at present, the PLQY

of such thin-shell samples are rarely reported to approach unity because of surface traps that are not well-isolated. Taking PLQY into account, the ideal samples should be QDs with small cores and thick shells (red star in Supplementary Fig. 26c). Based on the above discussion, we choose core/shell QDs with a 3.5 nm CdSe core and 7-8 monolayers of CdS shell as the model system. The up-conversion capability index η of this ideal QD sample is 0.05, which is one order of magnitude larger than that of Rhodamine 6G, a commonly used organic dyes (37 nm photoluminescence band-width and 30 nm Stokes shift, Supplementary Fig. 26b).

Determination of 'bright' and 'dim' states in single-dot photoluminescence measurements

The intensity threshold distinguishing 'bright' states from 'dim' states is set as three times the standard deviation of the background noise above the average background signals. The 'on' time fraction of a photoluminescence intensity trajectory is calculated as the fraction of time at 'on' state during the collection period. Because most of QDs display clear binary blinking behavior, the 'on' fractions are insensitive to the threshold value chosen.

Supplementary Figures:

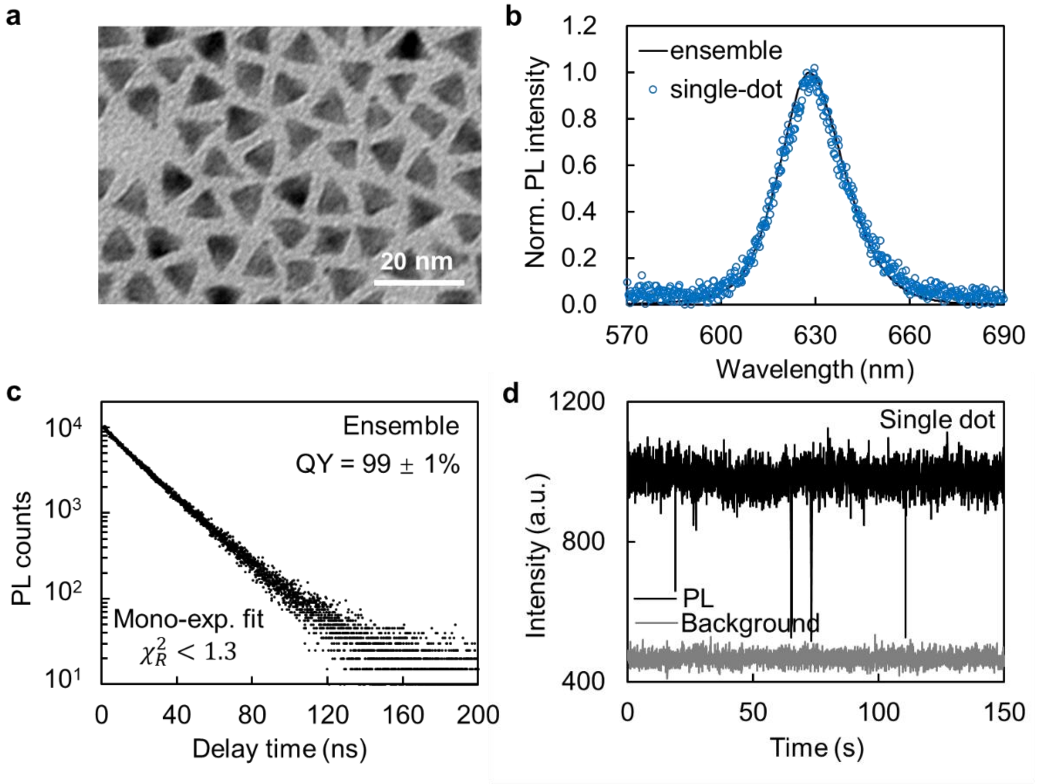

Supplementary Figure 1. Characterizations of the CdSe/CdS core/shell QDs with a 3.5 nm CdSe core and 8 monolayers of the CdS shell. a, Representative transmission electron microscopy (TEM) image of the QDs. **b,** Normalized photoluminescence (PL) spectra of the QDs in ensemble (black line) and single-dot (blue dot) levels. **c,** Photoluminescence decay dynamics of the en4semble QDs excited at 405 nm with a goodness-of-fit (χ_R^2) smaller than 1.3 for a mono-exponential fitting (lifetime being 24 ns). The absolute PLQY excited at 450 nm is 99±1% measured with an optical integral-sphere system. **d,** Representative photoluminescence intensity trajectory of a single QD (black) excited at 405 nm with background noises (grey). The bin time is 50 ms.

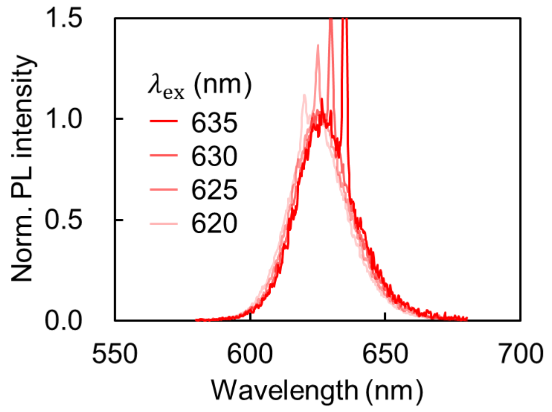

Supplementary Figure 2. Normalized PL spectra of QDs excited around the band edge. The normalized photoluminescence spectra with excitation wavelengths (λ_{ex}) ranging from 620 to 635 nm resemble each other with nearly no peak shift. The sharp peaks superposing on the photoluminescence spectra are the scattering of the excitation lights.

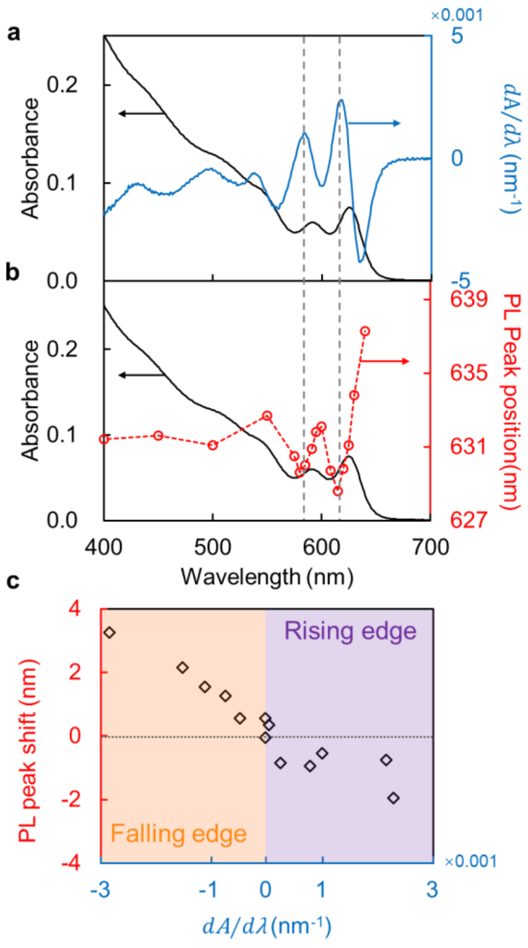

Supplementary Figure 3. Size inhomogeneity induced spectral difference between UCPL and DCPL of QDs in ensemble level. a, Absorption spectrum (black) and its first order derivative (dA/dλ, blue) of plain core CdSe QDs with relatively poor size homogeneity. **b,** Absorption spectrum (black) and photoluminescence peak positions (red) excited at different wavelengths. **c,** Correlation of photoluminescence peak shift and dA/dλ. The photoluminescence peak position of the QDs excited at 575 nm where dA/dλ = 0 was taken as a reference. Excited at the falling edge (red area) of the absorption spectrum where dA/dλ < 0, the photoluminescence peaks showed red-shift; while excited at the rising edge of the absorption spectrum where dA/dλ > 0 (blue area), the photoluminescence peaks showed blue-shift. Furthermore, the amounts of the shift are in good negative corrrelation

with the gradient of the absorption spectrum. Detailed discussions are shown in Supplementary Disscussion.

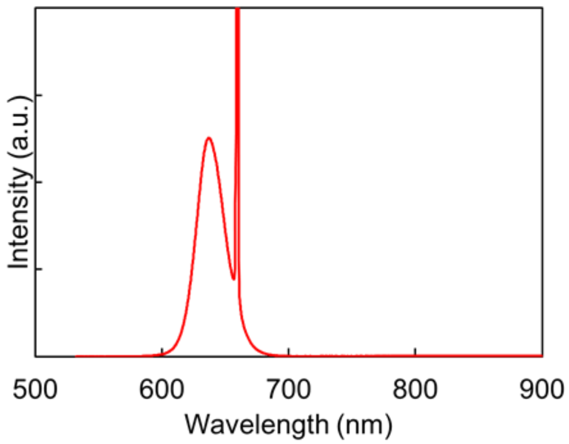

Supplementary Figure 4. Long wavelength part of the up-conversion photoluminescence spectrum. No defect-related emission is found in wavelength up to 900 nm.

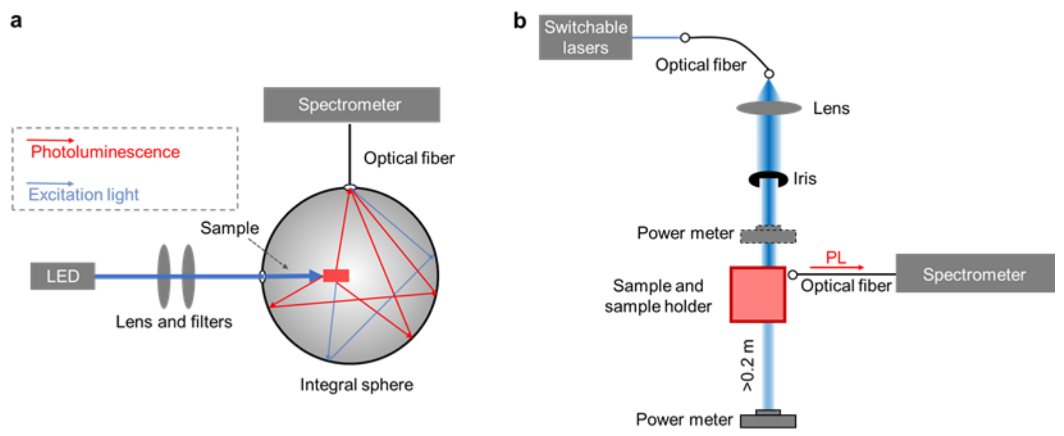

Supplementary Figure 5. Schematic diagrams of the setups for photoluminescence quantum yield measurements. a, Schematic diagram of the setup for absolute quantum yield measurements. LEDs with centre wavelength at 450 nm (or 532 nm) are directed to excite the sample in the integrating sphere. Photoluminescence and the residual excitation light exiting from the integrating sphere are collected by an optical fibre and recorded with a calibrated spectrometer. **b,** Schematic diagram of the relative quantum yield measurements. The excitation light is shaped with an optical fibre and an iris. The number of photons absorbed by the sample is determined with a power meter placed in front of and behind the sample. The distance between the sample and power meter is larger than 0.2 m when measuring the transmitted light power, in order to avoid the interference from emission light. The photoluminescence light is collected perpendicular to the excitation light and recorded with a spectrometer.

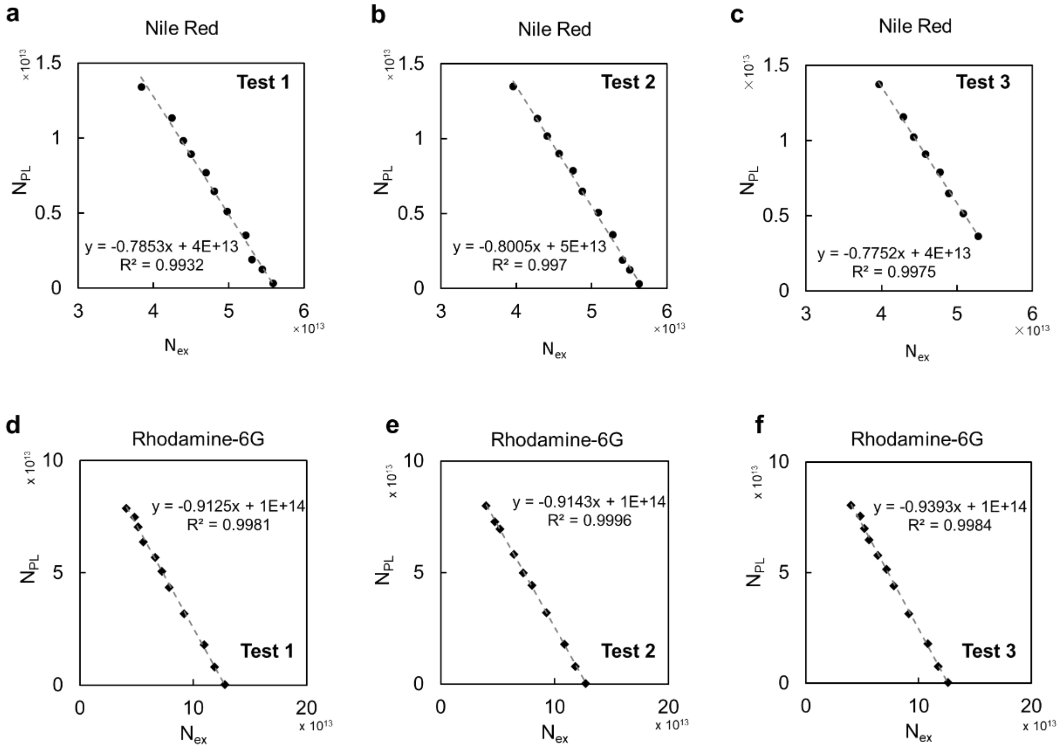

Supplementary Figure 6. **Absolute quantum yield measurements for standard dyes.** **a, b** and **c** are experimental results for the determination of quantum yield of Nile Red for three parallel tests. **d, e** and **f** are experimental results for the determination of quantum yield of Rhodamine-6G for three parallel tests. N_{PL} and N_t denote the photon numbers of the photoluminescence and residual excitation light. The grey dashed lines represent linear fits to the experimental data (Eq. S5). The absolute slopes of the fitting give the measured quantum yields.

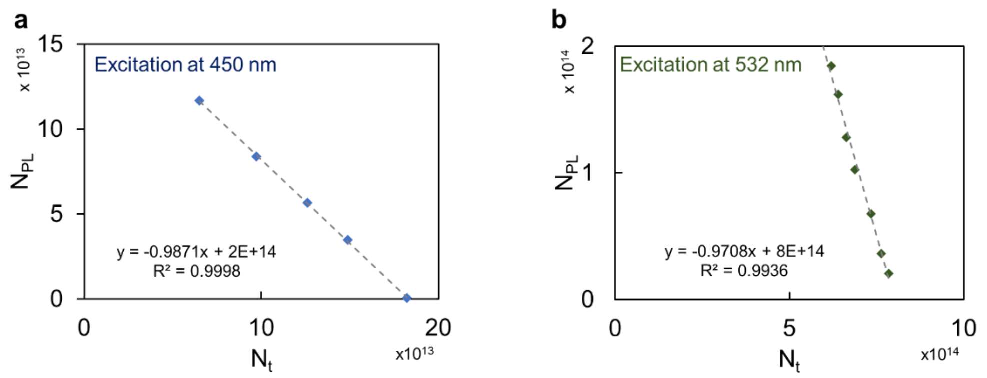

Supplementary Figure 7. **Absolute quantum yield measurements for quantum dots excited at a, 450 nm and b, 532 nm.** N_{PL} and N_t denote the photon numbers of the photoluminescence and residual excitation light. The grey dashed lines represent linear fits to the experimental data. The absolute slopes of the fitting give the measured quantum yields.

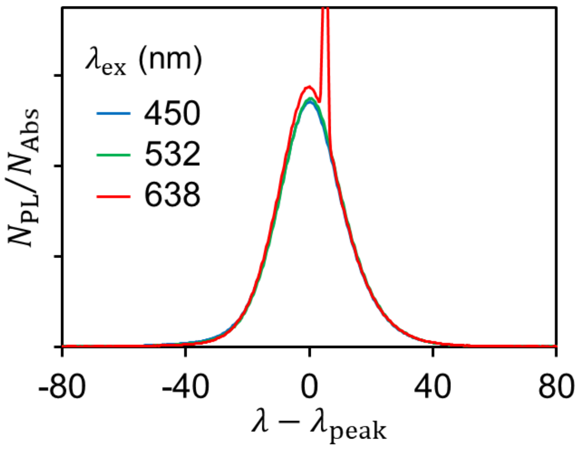

Supplementary Figure 8. Determination of PLQYs of QDs excited at different wavelengths. Displayed are photoluminescence spectra normalized by the number of photons absorbed by the QDs (N_{Abs}). N_{PL} is the number of emission photons at wavelength λ. The spectra are shifted to overlap with each other at the photoluminescence peak positions. The x-axis represents wavelength of the emission photon (λ) relative to the wavelength of the photoluminescence peaks (λ_{peak}). The excitation wavelengths are 450, 532 and 638 nm respectively. For calculating the PLQY excited at 638 nm, the UCPL spectrum is fitted with a Gaussian function to eliminate the influence of the excitation light. The integral areas of the normalized photoluminescence spectra should be proportional to the corresponding absolute PLQYs of the QDs excited at different wavelength. Since the absolute PLQY of the QDs excited at 450 nm is 99±1% measured by an optical integrating sphere, the absolute PLQYs excited at 532 and 638 nm were calculated as 99±1% and 100±1% respectively.

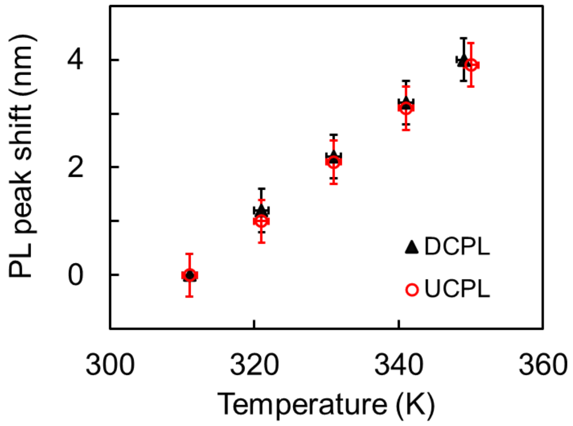

Supplementary Figure 9. Temperature dependent DCPL (black triangle) and UCPL (red circle) peak position shift. The photoluminescence peak positions at 311 K is taken as the references. The UCPL and DCPL show identical temperature dependence in the experimental temperature range.

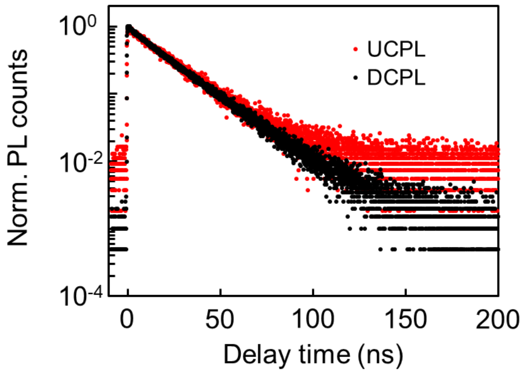

Supplementary Figure 10. UCPL and DCPL decay dynamics of ensemble QDs. The UCPL (red) and DCPL (black) decay dynamics are identical except their different background noise counts. The excitation power values for both DCPL and UCPL were low enough to avoid generation of multi-excitons.

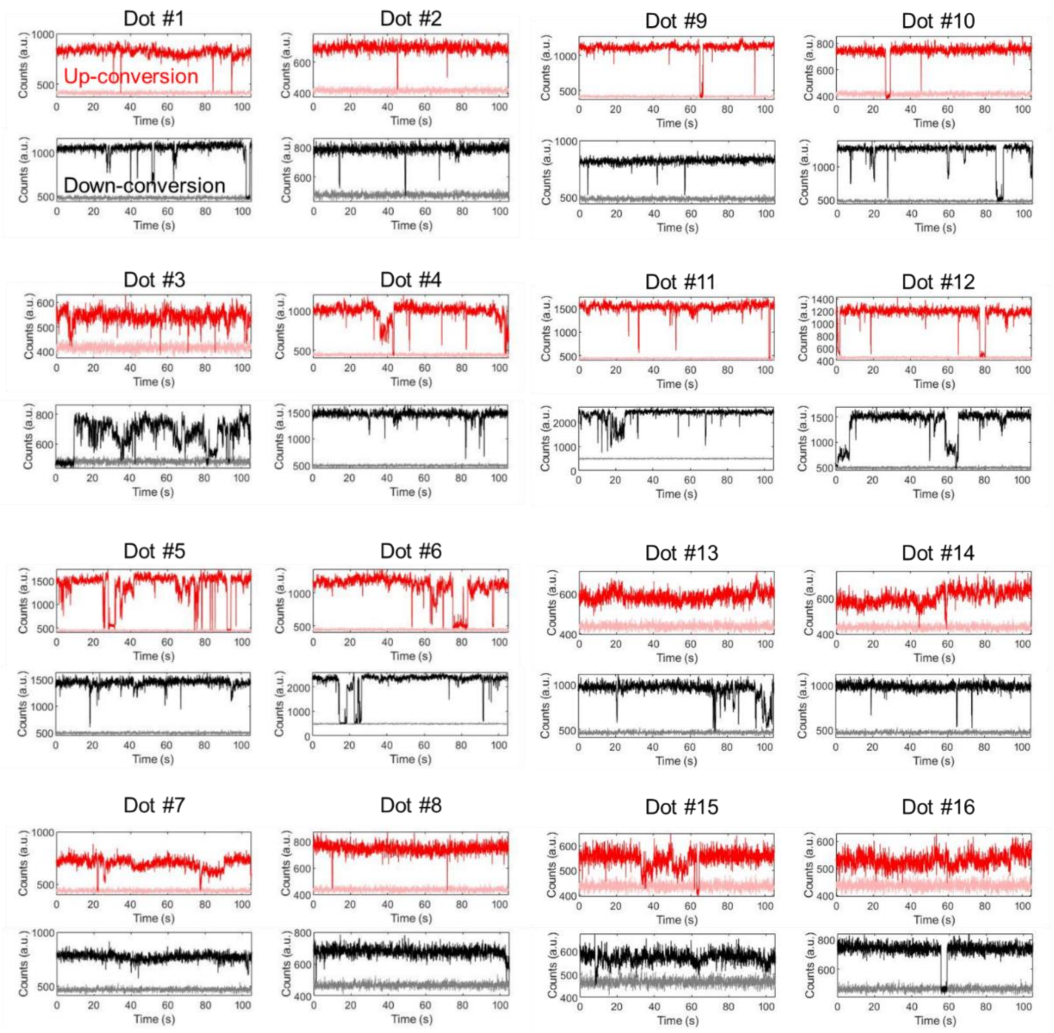

Supplementary Figure 11. Correlated UCPL (red) and DCPL (black) intensity trajectories of 16 single QDs. Corresponding background signals are shown in light colours. The excitation power densities were adjusted to ensure that the UCPL and DCPL intensities were similar for a given dot. (The detected UCPL intensity will be lower than DCPL because the long-wavelength part of UCPL was blocked by filters.) The bin time was set as 50 ms.

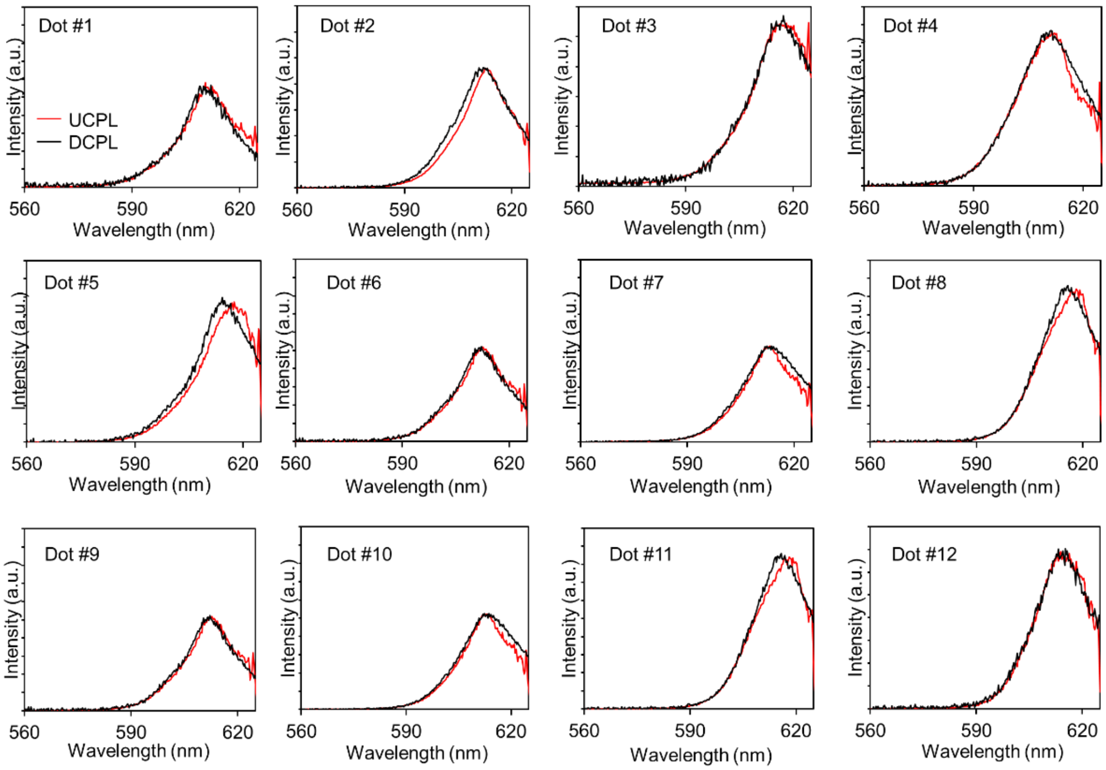

Supplementary Figure 12. Normalized steady-state UCPL (red) and DCPL (black) spectra of single QDs. The long wavelength tails of the UCPL spectra were blocked by the short-pass emission filters, which were used to cut-off the strong excitation lights. The influence of different filters used in UCPL and DCPL measurements are corrected.

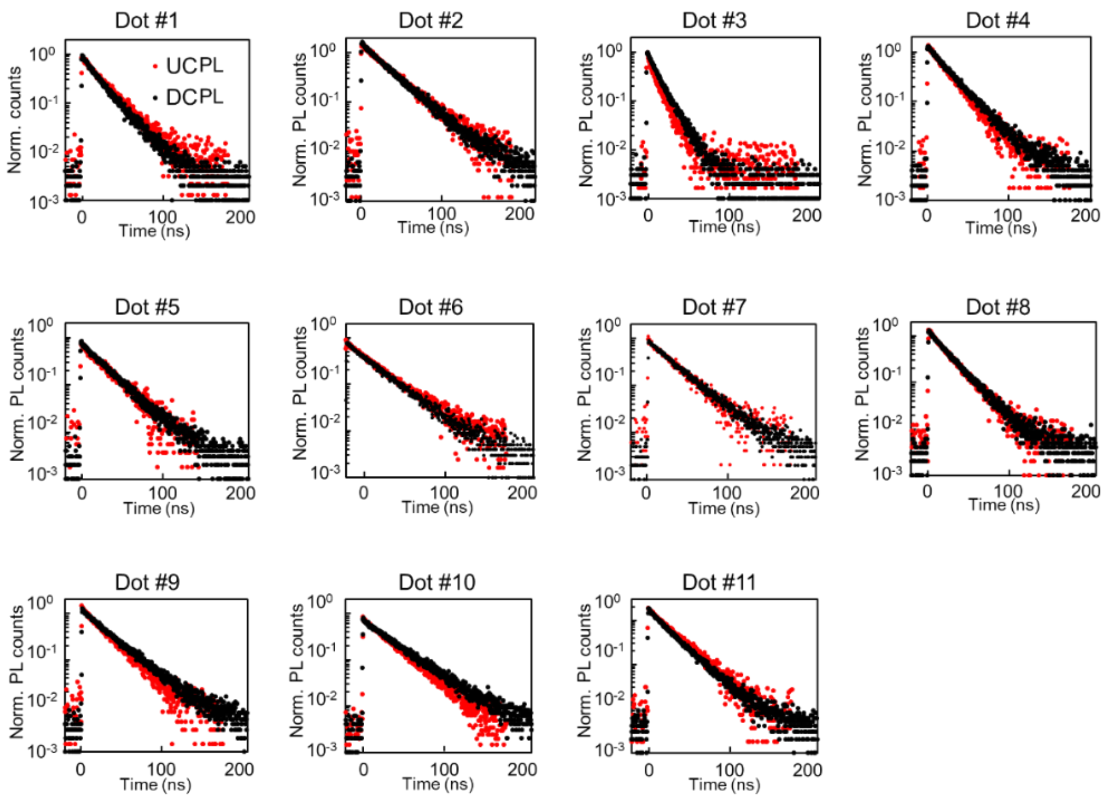

Supplementary Figure 13. Correlated UCPL and DCPL decay dynamics of single QDs. The background signals of UCPL are higher than that of DCPL because of longer integration time needed in UCPL measurements to accumulate similar number of photon-counts.

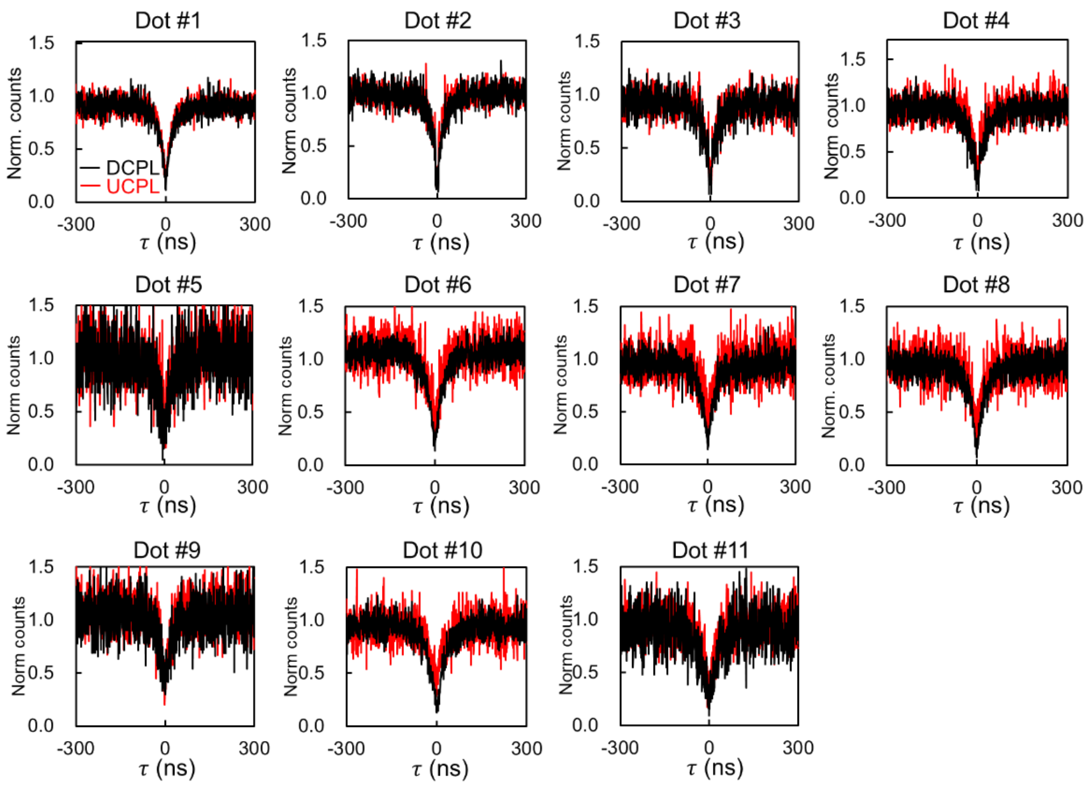

Supplementary Figure 14. Normalized UCPL (red) and DCPL (black) second-order photon correlations of single QDs measured with continuous-wave excitation. For each pair of UCPL and DCPL for a given QD, the same values of $g^{(2)}(0)$—the reading where delay time (τ) equals to zero—indicate the same quantum yields of the bi-exciton state, and the identical rising trends in each plot reveals the same decay dynamics of the bi-exciton state.

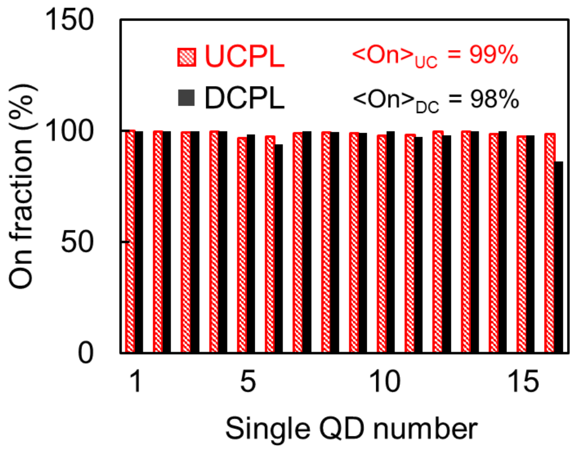

Supplementary Figure 15. ‘On’ time fractions comparison between UCPL and DCPL intensity time trajectories of single QDs. Single QDs show non-blinking behavior with average ‘on’ time fraction for UCPL and DCPL ($\langle On \rangle_{UC}$ and $\langle On \rangle_{DC}$) being 99% and 98%. The intensity time trajectories of single QDs are shown in Supplementary Fig. 11 and the definition of ‘on’ fraction is shown in Supplementary Discussion.

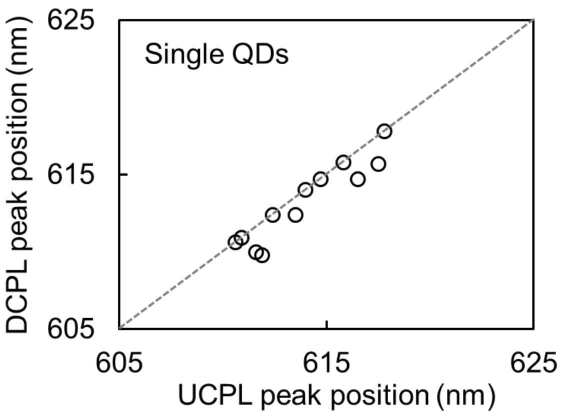

Supplementary Figure 16. Correlation of UCPL and DCPL peak positions of single QDs. The majority of data points are on the grey diagonal line, which indicates identical photoluminescence peak positions for up-conversion and down-conversion excitations. A few exceptions can be rationalized by considering the spectral diffusion. Once the spectrum diffuses towards long wavelength in UCPL measurements, the absorbance values of QDs at the excitation wavelength (long wavelength tail of the absorption spectrum) will increase. It leads to the increase of red-shifted photoluminescence ratio during the measurements and therefore cause the slight red-shift of the photoluminescence spectra under up-conversion excitation. The UCPL and DCPL spectra of single QDs are shown in Supplementary Fig. 12.

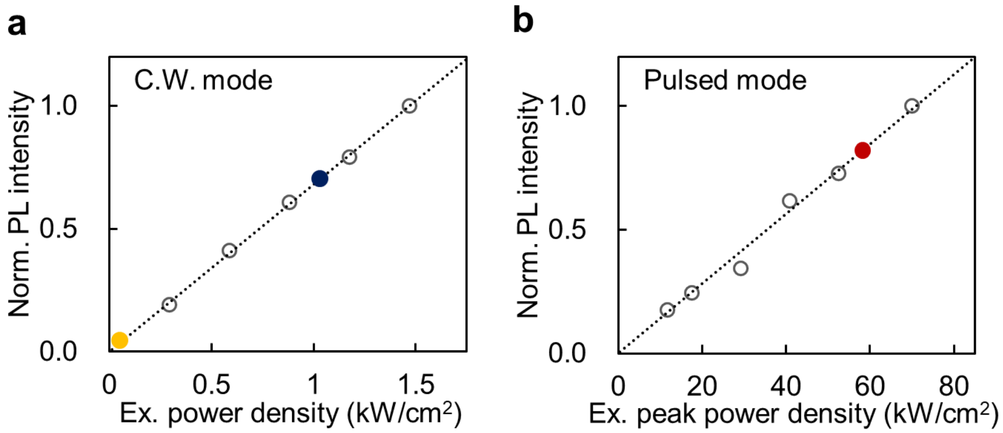

Supplementary Figure 17. Power dependent UCPL intensity for single-dot measurements with 633 nm laser excitation under (a) continuous-wave mode and (b) pulsed mode. The excitation power densities for UCPL intensity trajectory and decay dynamics measurements are marked in yellow and red respectively. The excitation power densities for steady-state UCPL spectrum and second-order photon correlation measurements are the same and marked in blue. The linear power dependent UCPL intensity confirmed that the up-conversion process in single-dot level is a single photon process.

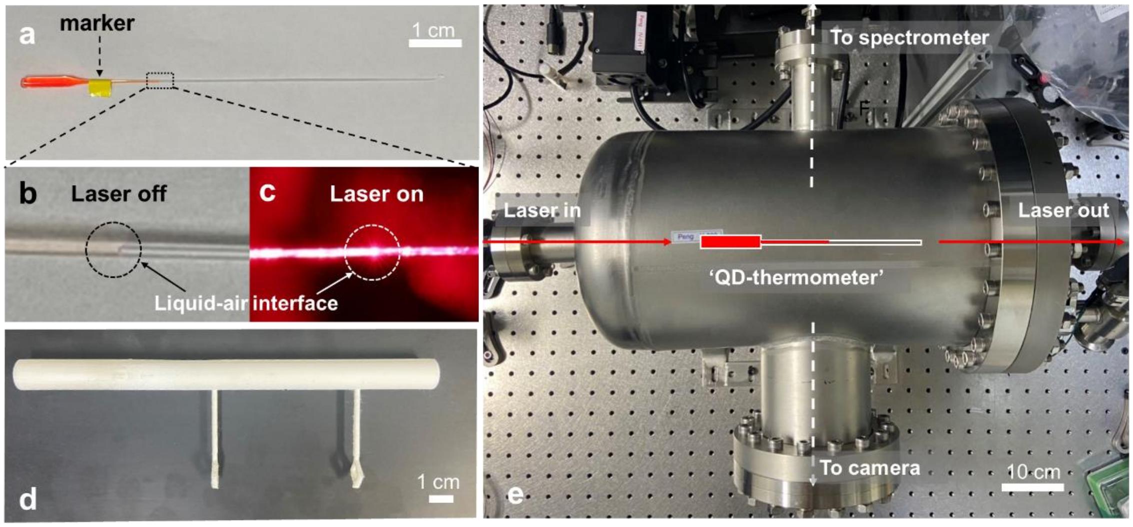

Supplementary Figure 18. Setup for the optical cooling experiments. a, Photograph of the 'QD-thermometer'. The quartz capillary tube with a tip reservoir is partially filled with QD solution and sealed at the end. A yellow marker with sharp edges is attached to indicate the volume change. **b** and **c** are magnified photographs of the 'QD-thermometer' in 'laser off' and 'laser on' stage. Dashed circles denote the liquid-air interfaces, whose positions were used to measure the volume change of the liquids during laser irradiation. **d,** Photograph of the diabatic holder made by 3D-printing with polylactic acid. **e,** Photograph of the vacuum chamber with the 'QD-thermometer' inside. Quartz windows were installed on the front, rear and side of the chamber.

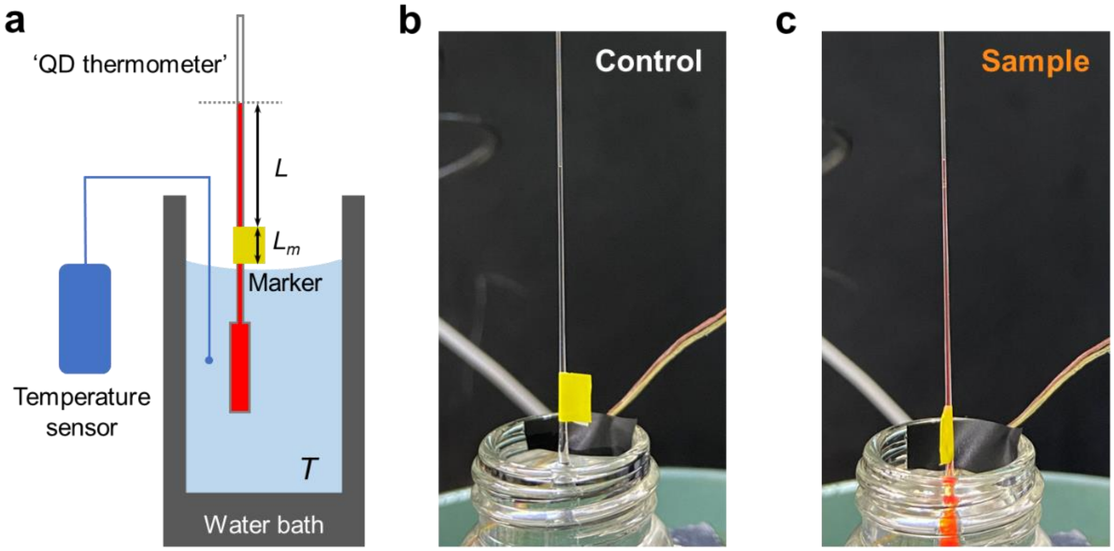

Supplementary Figure 19. Temperature calibration with a water-bath. a, Schematic diagram of the calibration setup. The length of the marker and the liquid column above the maker are denoted as L_m and L respectively. **b,** Photo of temperature calibration for the control specimen. **c,** Photo of temperature calibration for the QD sample. The quartz tube in **b** and **c** is the same one. The quartz tube is sealed before measurements.

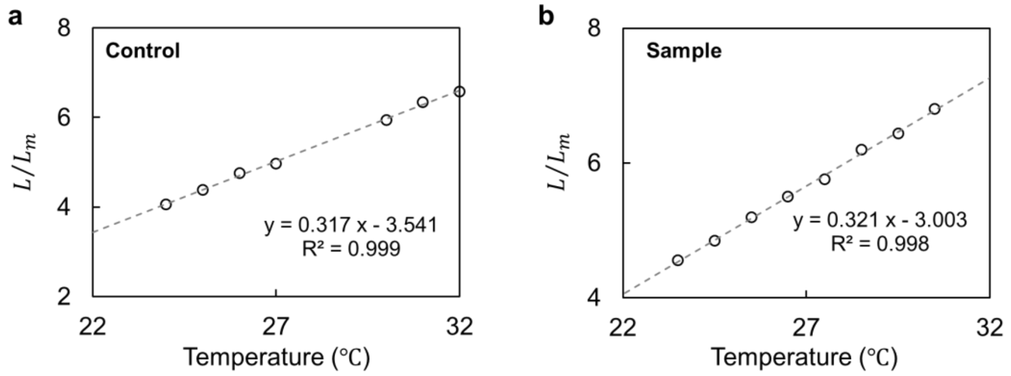

Supplementary Figure 20. Temperature calibration results for a, the control specimen and b, the QD sample. L_m and L are the length of the marker and the liquid column above the maker respectively.

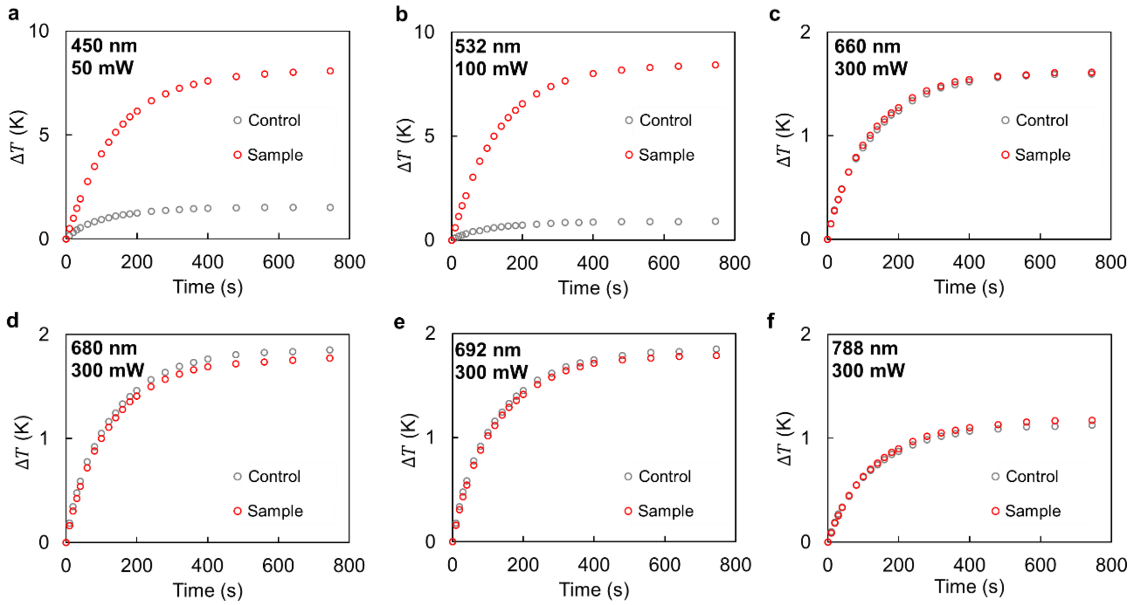

Supplementary Figure 21. Temperature change of the QD sample and control specimen under laser irradiation at different wavelengths. All results are the average values of three parallel tests. The temperature change at 671 nm is shown in Fig. 4c.

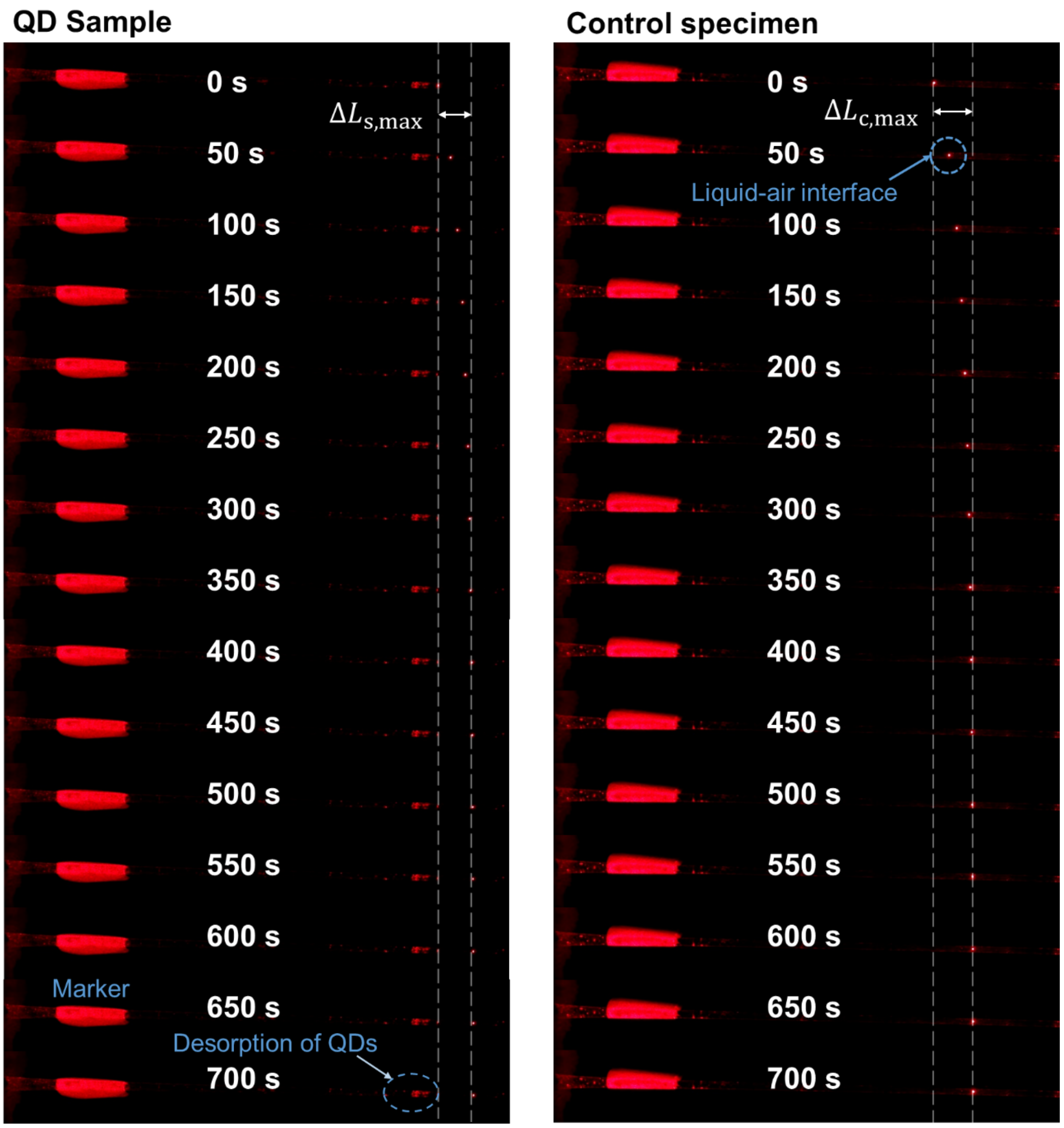

Supplementary figure 22. Photos of QD sample and control specimen under 671 nm laser irradiation at 300 mW. The red spots are liquid-air interfaces in the capillary tubes. $\Delta L_{s,max}$ and $\Delta L_{c,max}$ are the maximum position changes of liquid level in the capillary tubes of QD sample and control specimen respectively.

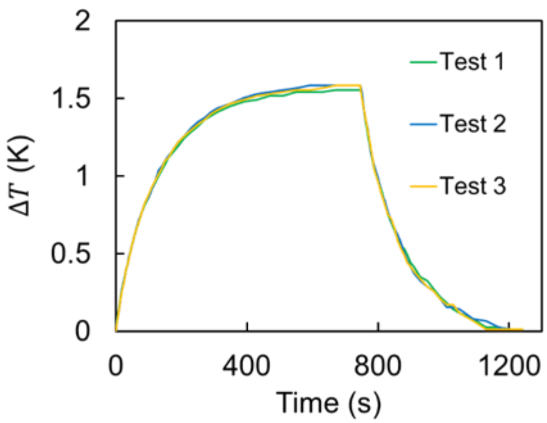

Supplementary Figure 23. Repeatability evaluation for the optical cooling experiments. Three parallel tests were carried out excited with 671 nm at 300 mW. Laser was turned on at 0 s and turned off at 750 s.

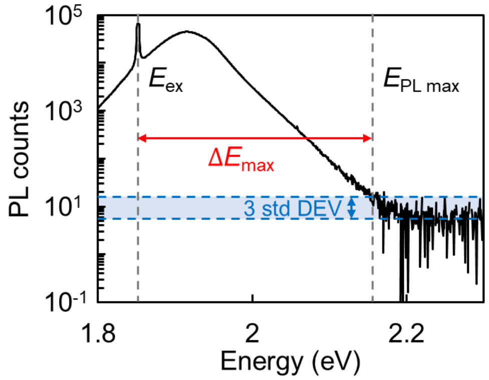

Supplementary Figure 24. Determination of maximum up-conversion energy gain (ΔE_{max}) of QDs. Displayed is a typical UCPL spectrum of CdSe/CdS core/shell QDs excited at 671 nm. ΔE_{max} is defined as the difference between the highest detectable photoluminescence photon energy ($E_{PL\ max}$) and the absorbed photon energy (E_{ex}). $E_{PL\ max}$ is defined as the photon energy where the photoluminescence intensity is 3 times the standard deviation (std DEV) of the background noise above the average background signals at the high energy side of the photoluminescence spectrum.

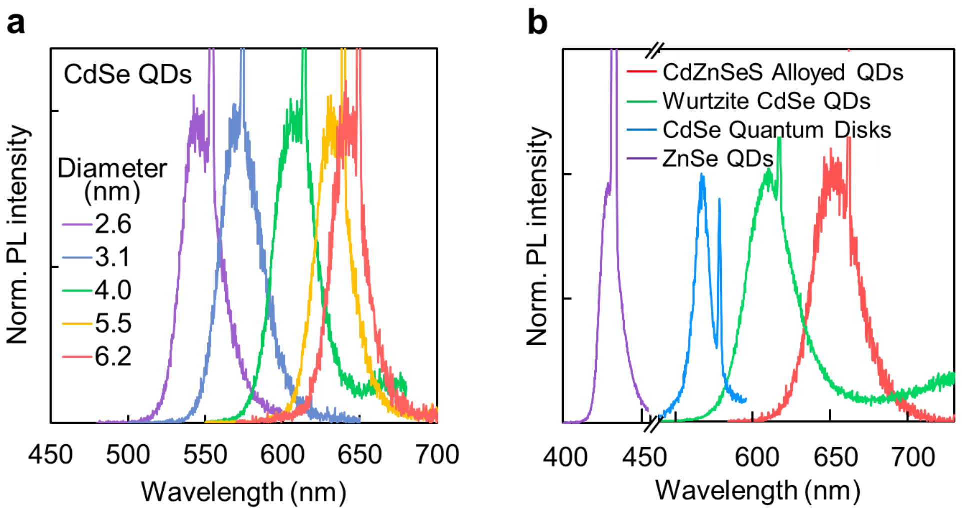

Supplementary Figure 25. UCPL of QDs with different sizes, crystal structures, morphologies and compositions. a, UCPL of zinc-blend CdSe QDs with different sizes ranging from 2.6 nm to 6.2 nm. **b,** UCPL of CdZnSeS alloyed QDs, wurtzite CdSe QDs, CdSe quantum disks and ZnSe QDs. The sharp peaks superposed on the photoluminescence spectra are from the scattering of excitation lights, whose wavelengths are longer than the corresponding emission peak positions.

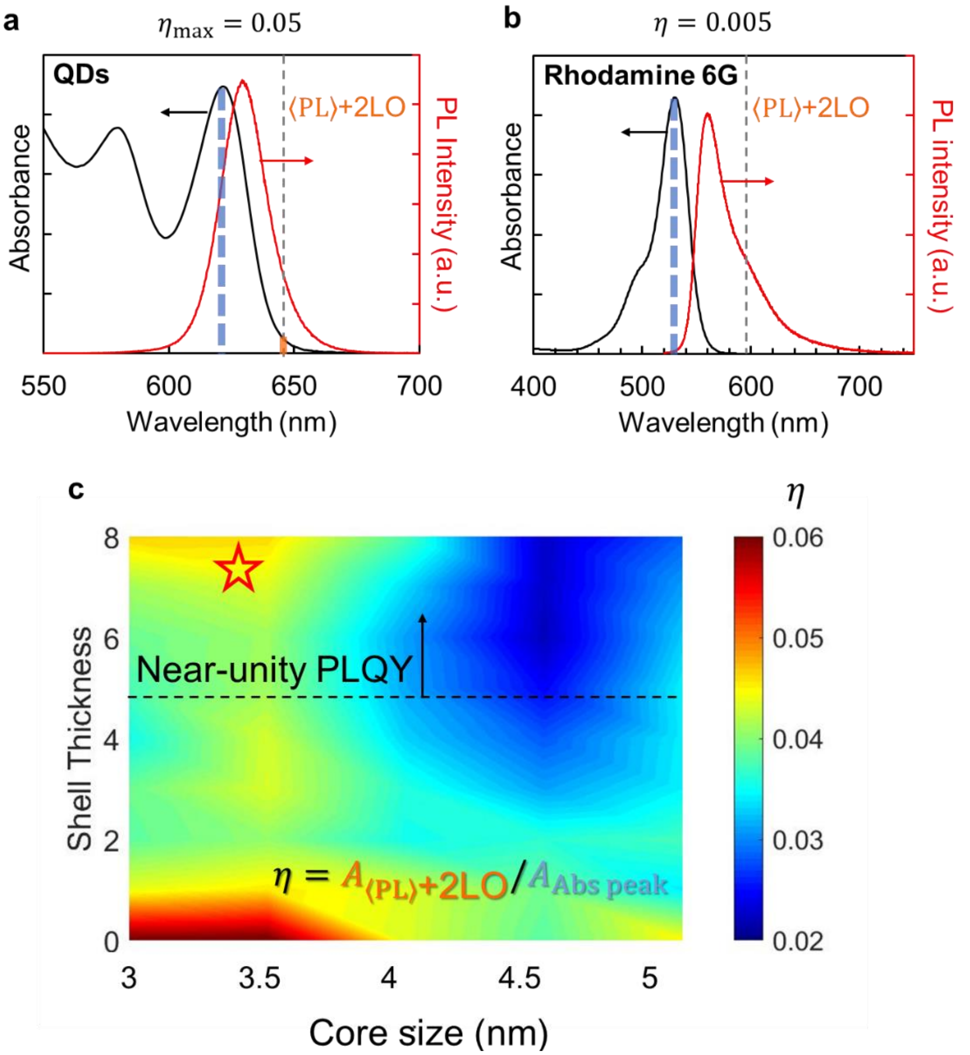

Supplementary Figure 26. Core size and shell thickness dependent up-conversion capability (η) of CdSe/CdS core/shell QDs. a, Absorption (black) and photoluminescence (red) spectra of typical QD solution. **b,** Absorption (black) and photoluminescence (red) spectra of Rhodamine 6G dissolved in ethanol. Grey dashed lines in **a** and **b** indicate the wavelength where the corresponding photon energy is 50 meV (twice the energy of CdSe optical phonons, 2LO) lower than the average photoluminescence photon energy. The absorbance at absorption peaks and grey lines are labeled as blue and orange thick lines. **c,** Up-conversion capability index (η) for CdSe/CdS core/shell QDs with different core sizes

and shell thicknesses. The definition of η is described in Supplementary Discussion. About 5 monolayers of the CdS shells are needed to achieve near-unity PLQY (black dashed line). The red star denotes the ideal core size and shell thickness with near-unity PLQY and the largest up-conversion capability index (0.05). For comparison, η of Rhodamine 6G is also calculated, and it's only 0.005.

Supplementary Reference:

1. Zhou, J. H., Zhu, M. Y., Meng, R. Y., Qin, H. Y. & Peng, X. G. Ideal CdSe/CdS core/shell nanocrystals enabled by entropic ligands and their core size-, shell thickness-, and ligand-dependent photoluminescence properties. *J. Am. Chem. Soc.* **139**, 16556-16567 (2017).
2. Sarkar, N., Das, K., Nath, D. N. & Bhattacharyya, K. Twisted charge-transfer process of nile red in homogeneous solution and in faujasite zeolite. *Langmuir* **10**, 326-329 (1994).
3. Fischer, M. & Georges, J. Fluorescence quantum yield of rhodamine 6G in ethanol as a function of concentration using thermal lens spectrometry. *Chem. Phys. Lett.* **260**, 115-118 (1996).
4. Lide, D. R. *CRC Handbook of Chemistry and Physics*. 84 th edn, (CRC Press, 2004).
5. Poles, E., Selmarten, D. C., Micic, O. I. & Nozik, A. J. Anti-Stokes photoluminescence in colloidal semiconductor quantum dots. *Appl. Phys. Lett.* **75**, 971-973 (1999).
6. Wang, X. Y. *et al.* Photoluminescence upconversion in colloidal CdTe quantum dots. *Phys. Rev. B* **68**, 125318 (2003).
7. Rakovich, Y. P. & Donegan, J. F. in *Semiconductor Nanocrystal Quantum Dots Synthesis, Assembly, Spectroscopy and Applications* (ed Andrey L. Rogach) Ch. 9, 257-275 (Springer-Verlag, Vienna, 2008).
